# Climate-specific health literacy in health professionals: an exploratory study

**DOI:** 10.3389/fmed.2023.1236319

**Published:** 2023-10-20

**Authors:** Lorenz Albrecht, Lydia Reismann, Michael Leitzmann, Christine Bernardi, Julia von Sommoggy, Andrea Weber, Carmen Jochem

**Affiliations:** ^1^Department of Epidemiology and Preventive Medicine, University of Regensburg, Regensburg, Germany; ^2^Department of Epidemiology and Preventive Medicine, Medical Sociology, University of Regensburg, Regensburg, Germany; ^3^University Children’s Hospital Regensburg (KUNO), University of Regensburg, Klinik St. Hedwig, Regensburg, Germany

**Keywords:** climate-specific health literacy, health professionals, physicians, nurses, climate change

## Abstract

**Background:**

Health professionals such as physicians and nurses may play an important role in the transformation process towards a healthy, sustainable and climate-sensitive society. However, little is known about their climate-specific health literacy. This study aimed to assess knowledge regarding climate change and its impacts on health and climate-specific health literacy in health professionals.

**Methods:**

In July/August 2022, a cross-sectional, questionnaire-based study was carried out at the University Hospital Regensburg, Germany, to assess climate-specific health literacy in nurses and physicians from various clinical specialties. Descriptive and exploratory statistical analyses were performed.

**Results:**

The study population consisted of 142 participants (57.7% women; response rate: 24,7%). Most participants (93%) considered climate change to be highly relevant. However, only 12% of respondents stated to be very well informed regarding the general consequences of climate change. Although 57% of all participants had never mentioned climate change in relation to health to their patients, participants with higher levels of knowledge regarding the effects of climate change were more likely to mention it compared to those with lower levels of knowledge. The most frequently stated obstacle to integrate the topic of climate change in clinical work was lack of time during work (79%), not enough information (42%) and lacking materials (39%). Differences between health professions were apparent.

**Conclusion:**

The results of our survey suggest that the current state of climate-specific health literacy differs between different groups of health professionals. There is a need to improve health professionals’ levels of climate-specific health literacy and to increase the potential in interprofessional cooperation regarding planetary health.

## 1. Introduction

Climate crisis is seen as the biggest global health threat in the 21st century (1). There is a broad scientific consensus about the urgency of transformative action to limit climate change (2). Every year, 150,000 deaths could be avoided by more ambitious climate protection in Germany (3). Climate-specific health literacy might be crucial for climate change mitigation and improving individual and planetary health (4). According to Reismann et al., the concept of climate-specific health literacy encompasses knowledge about the present and long-term health risks of climate change and knowledge about the health co-benefits of health promoting behaviours related to climate change. Moreover, the concept includes emotional integration of knowledge and feelings of concern related to climate change and health and the ability to implement this knowledge into action and climate-friendly behaviour (4). Planetary health is defined as “the health of human civilization and the natural systems on which it depends” (5).

The health care sector, aiming at protecting the human health, is a relevant player in the needed transformation process towards a climate sensitive and thus healthier society as it has an impact on climate and environment, and at the same time supports society in health and disease. Nurses and physicians, who have important roles within the health care sector, regularly rank first in relevant international surveys in regard to society’s trust in them and their recommendations (6–9). Since the climate crisis has numerous consequences for health, health professionals nowadays have a great opportunity and responsibility to make appropriate use of the trust placed in them to promote climate-sensitive societies (10). In their daily work routine, health professionals have a lot of opportunities to address climate change and health. This can enable patients to become more climate-sensitive themselves and thus protect the climate and be better prepared to deal with the inevitable impacts of climate change (4). Furthermore, health professionals can contribute to health promotion and sustainable development through their own behaviour, e.g., healthy and sustainable nutrition and active transportation. These enumerated behaviours require the aforementioned climate-specific health literacy.

Therefore, the climate-specific health literacy among health professionals plays an important role in tackling climate crisis and in coping with climate crisis related impacts. It is particularly important that different health professionals work together on this issue and create synergies. However, until now, little is known about the climate-specific health literacy of health professionals. To our knowledge, there has been no study in the German-speaking world that has investigated the climate-specific health literacy among health professionals (11). What is the current state of climate specific health literacy among health professionals? Does the status differ between the different health professions? What are obstacles and what is conducive to the development and exercise of this skill? To answer these questions, we conducted the following study at a German university hospital.

## 2. Materials and methods

### 2.1. Setting and time

During July and August 2022, we conducted a cross-sectional, questionnaire-based study on climate-specific health literacy in health professionals (nurses and physicians) of the University Hospital Regensburg (estimated number of people who were invited to participate n = 575), Germany. The link to the online questionnaire was distributed by email, by posters and by direct talks to employees of all levels of seniority aged at least 18 years.

All participants gave informed consent to the anonymous and voluntary data collection. The study protocol was approved by the ethics committee (University of Regensburg), approval number 22–2893-101.

### 2.2. Study instrument

We designed an online-based questionnaire in German language with 22 items using LimeSurvey Professional (12). The questionnaire consisted of closed-ended questions with single (yes/no) or multiple-choice items or 5-point Likert-type items. 13 questions originated from a questionnaire that was developed based on scientific literature by Reismann et al. (4). These questions have been used before and showed good face validity (4, 13). Another three questions from this tool were adapted to our target group and six new self-generated questions were added to the survey: status quo, barriers, and enabling factors of climate-specific health literacy of health professionals. The English version of the questionnaire used in the present study is shown in Supplementary material 1.

The questionnaire consisted of four sections, namely: (1) Demographic characteristics (four questions on gender, age, job (physician or nurse) and medical discipline); (2) Self-assessed awareness and knowledge of climate change in general and willingness for climate friendly behaviour (seven questions); (3) Climate change and health (four questions); (4) Climate change mitigation in the healthcare system and climate-sensitive health advice (seven questions). The questionnaire was pilot tested in health professionals (*n* = 25) and was revised accordingly. Reporting was voluntarily for each question.

### 2.3. Data analysis

We carried out descriptive statistics and exploratory data analyses. For categorical data, we computed frequencies and proportions. Medians and interquartile ranges were computed for continuous data. We used multivariate regression analyses to examine relations of gender, age, job and self-assessed knowledge regarding climate change. We employed ordinal logistic regression for responses on the ordinal scale (e.g., Likert scale) and logistic regression for binary responses. We adjusted all regression models for the potential confounding variables of age and gender. All tests were two– sided and a value of *p* of <0.05 was deemed statistically significant. R (statistical software version 4.2.1) was used for the statistical analyses (14).

## 3. Results

### 3.1. Demographic characteristics

Of the 575 health professionals receiving the link to the questionnaire, 180 filled in the online questionnaire. Of these, 38 were excluded due to missing information on gender, age, and job leading to an analytic sample of 142 participants (57.7% women; response rate 24,7%), including 84 physicians and 58 nurses. Most participants worked in internal medicine (34%), followed by surgery (30%) and anaesthesia (13%). While physicians and nurses from internal medicine and surgery were almost equally represented (52% vs. 48% / 53% vs. 47%), mainly physicians took part from anaesthesia (94% vs. 6%). Participants were aged 18 to 63 years. The median age was 38 years in physicians and 45 years in nurses (Table 1).

**Table 1:** Demographic characteristics of respondents according to job (physician/nurse) (*n* = 142).

	Physicians (*n* = 84)	Nurses (*n* = 58)
Gender		
Women (n = 82)	35	47
Men (n = 60)	49	11
Non-binary (n = 0)	0	0
Age		
Median (25th percentile – 75th percentile)	38.0 (31.0–45.0)	44.5 (29.0–51.8)
Specialization		
Internal Medicine (*n* = 48)	25	23
Surgery (*n* = 43)	23	20
Anesthesia (*n* = 18)	17	1
Radiation/ Nuclear Medicine (*n* = 9)	5	4
Others* (*n* = 22)	13	9
NA (*n* = 2)	1	1

Others*: Ear/ Nose/Throat Medicine (*n* = 6), Ophthalmology (*n* = 6), Dermatology (*n* = 5), Neurology (*n* = 3), Pediatrics (*n* = 2).

### 3.2. Self-assessed awareness and knowledge of climate change in general and willingness for climate friendly behaviour

**Figure 1:**
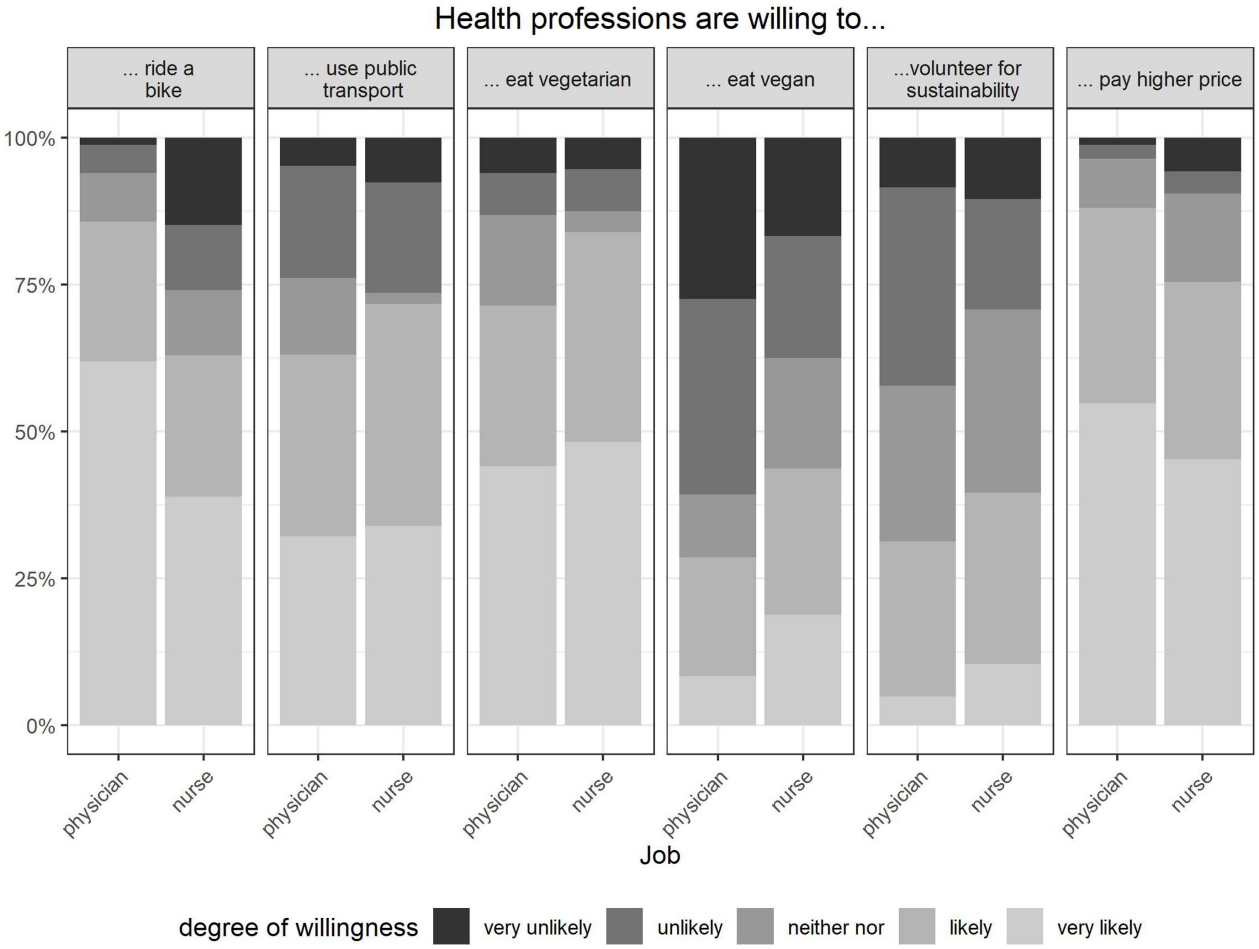
Willingness to engage in climate-friendly behaviour in different behaviour-items by physicians and nurses, Percentage of respondents who self-reported their willingness to implement health co-benefits in everyday life. Multiple choice was possible, *n* = 142.

The majority of the participants (93%) considered climate change to be (very) important. Self-assessed knowledge about the consequences of climate change was stated to be very good by 12%, good by 45%, moderate by 40% and rather low by 3%, respectively. Physicians reported higher levels of willingness to pay higher prices for climate-friendly products (*p* < 0.05) and to ride a bike (*p* < 0.05) compared to nurses (Figure 1). Compared to men, women (physicians and nurses) were more willing to eat a vegetarian (*p* < 0.001) or vegan (*p* < 0.01) diet. Also, participants with higher level of self-assessed knowledge regarding the general consequences of climate change were more likely to eat vegetarian diet (*p* < 0.05), and to volunteer for sustainability (*p* < 0.05) compared to participants with lower levels of self-assessed knowledge.

Most participants (81,9%) considered knowledge about health co-benefits followed by knowledge about health benefits (81,8%) as very likely or likely to increase their willingness for climate-friendly behaviour. Acting together in the peer group or acting as role models was identified as a reinforcing factor by the majority of participants (70 and 65%, respectively).

### 3.3. Climate change and health

Most of the participants affirmed climate change as a (very) likely cause of global health problems and a (very) likely risk factor for their own and their patients’ health (83, 71, and 77%, respectively). Compared to physicians, nurses more frequently reported climate change as a (very) likely cause of global health problems (90% vs. 80%) and as a (very) likely risk factor related to the health of their own patients (85% vs. 73%) and their own health (88% vs. 58%).

**Figure 2:**
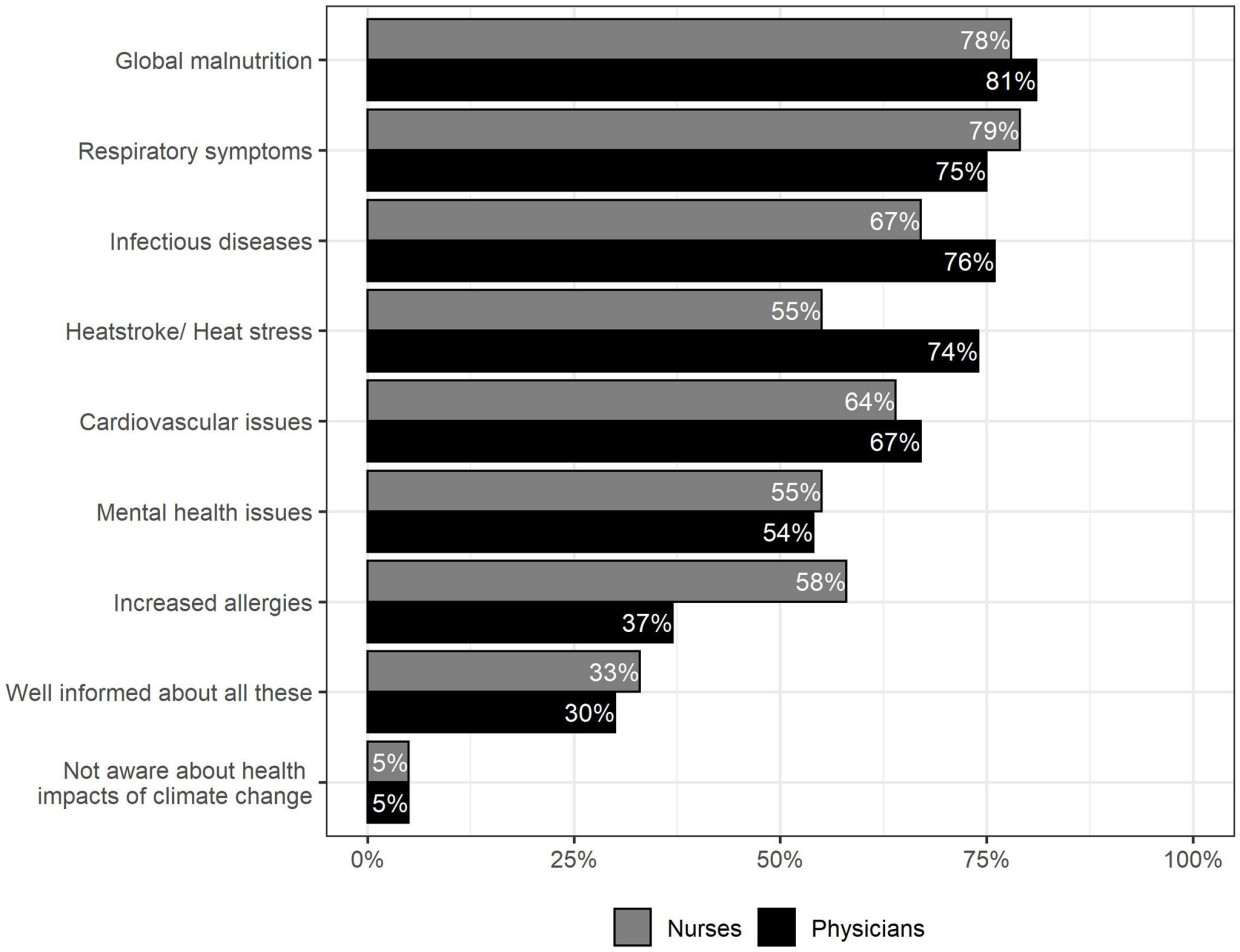
Participants’ awareness of health-related effects of climate change, Multiple choice was possible, *n* = 142.

Physicians affirmed they had already heard about global malnutrition (81%), infectious diseases (76%), respiratory symptoms (75%) and heatstroke (74%). Nurses had most often heard of respiratory symptoms (79%), global malnutrition (78%), infectious diseases (67%) and cardiovascular issues (64%) (see Figure 2).

### 3.4. Climate change mitigation in the healthcare system

When asked if medical professionals should be committed to climate change mitigation to ensure long-term health, 75% of physicians responded, “yes definitely” or “rather yes,” while 18% chose “neither nor” and 6% “rather no.” Nurses’ responses were similar with 69, 28, and 3%, respectively.

**Figure 3:**
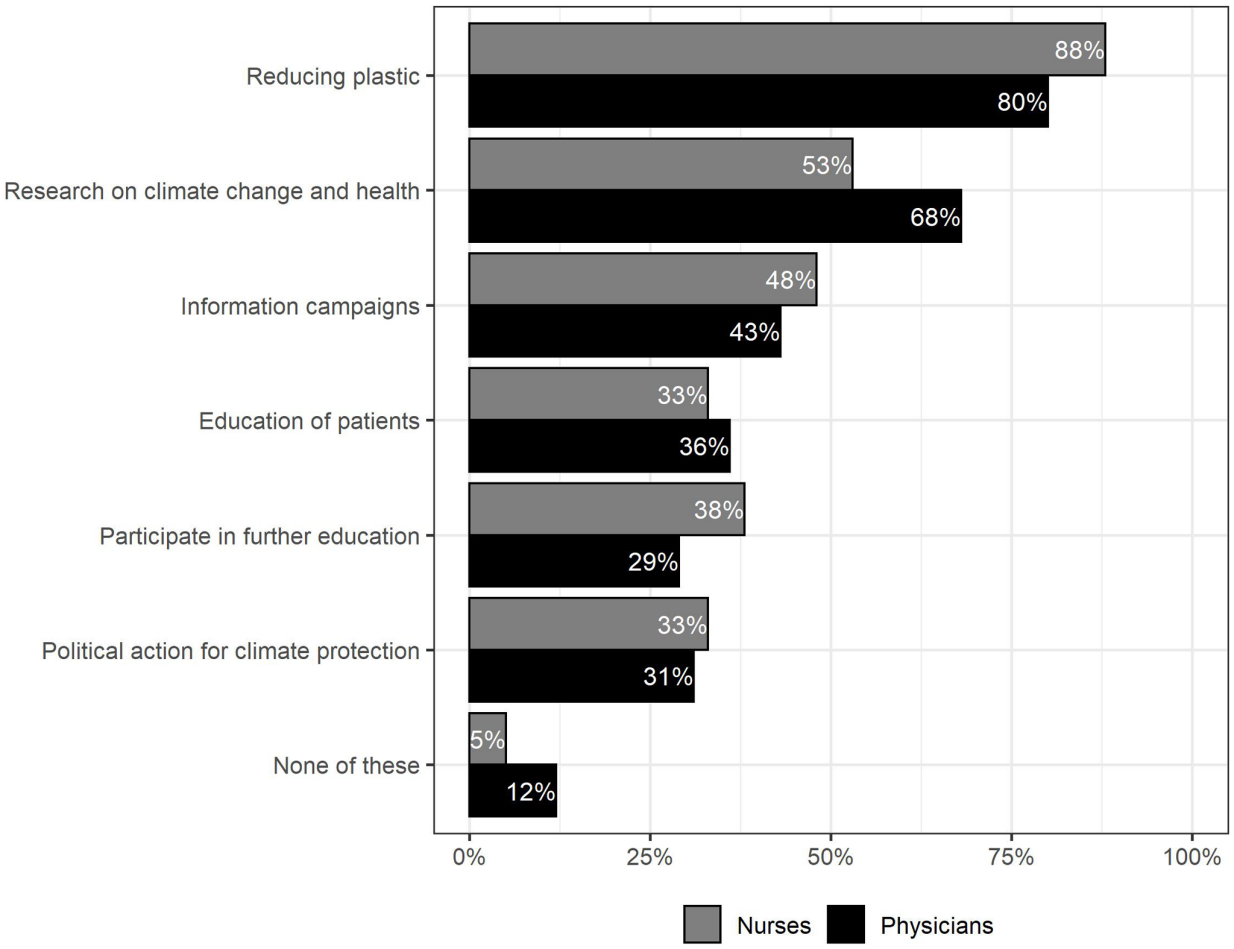
In what way do you think medical staff should support sustainability and preventive health protection? Multiple Choice was possible, *n* = 142.

Health workers stated that medical staff should be committed to sustainability in form of “reducing plastic” (80% of physicians, 88% of nurses), “research on climate change and health” (68% of physicians, 53% of nurses), “information campaigns” (43% of physicians, 48% of nurses) and “education of patients” (36% of physicians, 33% of nurses), see Figure 3.

### 3.5. Climate-sensitive health advice

Most participants (57%) never mentioned climate change in relation to health in their clinical context. Participants with higher levels of knowledge regarding the effects of climate change were more likely to mention it (49%) compared to those with lower level of knowledge (34%). While surgery physicians were less likely to mention climate change in relation to health in contrast to internal medicine physicians (21% versus 57%), surgical nurses were more likely to mention the connection (60%) than internal medicine nurses (52%).

“Time to be able to address the topic in my daily work” (79% of physicians and nurses), “Information about the scientific background of the topic” (36% physicians / 52% nurses), “Materials, that I can use to explain the topic” (35% physicians/ 47% nurses) was selected as helpful to better educate the patients about climate change and health.

## 4. Discussion

The key findings from our work were that the participating health professionals viewed the climate crisis as an important issue and recognized the connection between the climate crisis and health. Furthermore, the majority of health professionals surveyed did not have sufficient knowledge regarding climate-specific health literacy. Obstacles to develop and practice these skills are primarily a lack of time and a lack of information and materials, with nurses citing the latter two most often. Components of climate-sensitive health literacy differ across health professions. In perspective, there is scope for improvement, for example through more education and better information material. These findings are in line with previous study results, where large proportion of participants also reported not being well informed about the consequences of the climate crisis or being prevented by, e.g., lack of time from counselling their patients (15, 16).

Study participants were ready to adopt various climate-friendly behaviours for example, riding a bicycle or using public transport instead of driving a car, eating a vegetarian diet and paying a higher price for climate-friendly products. These actions affect the health professionals individually in their daily lives and, in part, already actively protect the climate and their own health as health co-benefits.

However, the fact that a large proportion of health professionals themselves have never addressed the issue of climate crisis in relation to their patients’ health in their clinical work indicates that it is important to educate health professionals that by doing so they can positively impact their patients and support and improve their climate-specific health literacy.

### 4.1. Climate-specific health literacy

The aforementioned concept of climate-specific health literacy, with its various components mentioned above, can be an important tool for taking climate action. If health professionals were themselves well trained in climate-specific health literacy, and had good resources to educate patients about relationships between the climate crisis and health, great potential could be tapped. In their study, Reisman et al. were able to show that patients who were informed by physicians about the consequences of the climate crisis on their health had higher awareness about climate related health risks and behaved in a more climate-sensitive manner (4). In a study in university students, Weber et al. (13) confirmed this assumption and showed that students who reported to be better informed about the relationship between the climate crisis and health were consistently more willing to engage in climate-friendly behaviours and actually did so. Other studies also indicated that the climate crisis becomes more tangible for people when the consequences for their health are brought to the fore: People are more motivated to take climate action when they know about the impact on their own health (17, 18).

Information about the climate crisis has a greater reach when it is health-related than other types of information about the climate crisis (18). In line with previous data, our findings suggest that the climate crisis is still seen as a global health problem rather than a problem for one’s own health or for the health of one’s own patients (4, 18). This finding underlines the importance of direct education of patients by health professionals, as both trusted advisors and role models, regarding the impact of the climate crisis on health.

As the climate crisis is no longer a remote optional event that can be prevented in its entirety, and thus numerous impacts on human health are already being experienced today (19), health professionals should not only demand climate protection and mitigation measures, but also actively invest in the adaptation of their patients and educate them about health risks through appropriate counselling (20).

### 4.2. Interprofessional training and team collaboration

The results of our survey suggest that the current state of climate-specific health literacy differs between the two different groups of health professionals under study. These differences, e.g., in risk perception and counselling, are not yet fully investigated. A potential explanation could be that the respective professional groups spend different amounts of time with patients and thus have different amounts of time to talk about the topic. Another explanation could be that the topic has been taught to different degrees so far. To our knowledge, the topics of climate crisis and health/planetary health have not yet been comprehensively integrated into the compulsory curricula of nursing and medical students. Nevertheless, there is great potential in this area, for which basic ideas already exist (21). Further research is needed to explain the differences in climate-specific health literacy among the professions to then best develop and promote them subsequently. Furthermore, we see great potential in interprofessional cooperations. There is consensus that interprofessional working among health professionals is important for safe and successful patient care (22–24). This may also be the case when addressing the issue of the climate crisis and health/ planetary health. It is already within the basic understanding of this discipline that planetary crises can only be confronted together in a transdisciplinary manner, as their magnitude and significance involve numerous fields (25). For this reason, too, successful interprofessional working can and should be fostered through appropriate promotion of the necessary skills in education and training (24). One possible interprofessional activity is through simulation, in which the different health professional learners carry out consultations together addressing climate related health problems and education with simulated patients, followed by debriefing and reflection (26).

### 4.3. Barriers and promotion to climate-specific health literacy for health professionals

Looking for the main obstacles for health professionals including climate-specific health advice in their work, our questionnaire mainly revealed the three obstacles lack of time, lack of information and materials.

Nurses are working under great time pressure, which has been exacerbated during the COVID-19 pandemic, and therefore do not have time to educate their patients on additional topics (27, 28). Nevertheless, health care professionals are committed to protecting human life (29) and to participate in the preservation of the natural foundations of life in view of their importance for human health (30). Recommendations for effective policy-level engagement already exist for staff with limited time (31). To integrate climate sensitive health advice in communication with patients, measures are needed to support nurses and physicians and to create opportunities to perform this important task. One approach could involve health insurance providers in Germany compensating physicians to provide climate-sensitive health advice, although this would probably not solve the time problem.

It would be preferable if climate change resilience education became part of all health professional curricula and was illustrated with relevant and everyday clinical examples, e.g., in form of burdens and risks of vulnerable groups. An approach already occasionally implemented by physicians takes patients’ concerns as an opportunity to include planetary health references in the consultation where appropriate (32).

Further research is needed to develop and evaluate methods that enable health professionals to provide climate sensitive health advice despite time constraints.

Many participants in our study stated that they need more knowledge and materials to better educate patients about the topic. Therefore, we agree with the demands of Guzman et al. as well as the Lancet Policy Brief Germany 2019 & 2021 (33) and the resolution of the 126th German Medical Congress (34) that the topic of climate crisis and health/ planetary health should be an obligatory part of the education and training of health professionals (35). It has been shown that health professionals (15) and students (36) are interested in this topic and appreciate an integration in their education. Experiences, ideas and concepts of what planetary health education could look like have already been outlined in studies and models (35, 37–40).

### 4.4. Strengths and limitations

As one of the first studies in a German maximum care hospital, we were able to survey physicians and nurses and compare the answers depending on profession and specialty. Although fewer nurses participated in the questionnaire, the proportions are better balanced than in previous studies.

Although our sample size is not very large it was possible to identify trends and to derive first exploratory results. As in all self-report methods, the answers to our questionnaire might be affected by social desirability.

While the study provides initial exploratory quantitative results, further multicenter and multinational mixed methods studies including qualitative methods are needed.

Selection bias could be present if mainly individuals interested in the topic of climate change participated in the study. This might distort our results to the extent that climate-sensitive health literacy appears to be more developed than it actually is. Our questionnaire focused mainly on climate-specific knowledge, thus leaving out some relevant planetary health references. A broader approach that also focuses on other planetary crises in addition to the climate crisis would also be very important and should be focused on in future work.

## 5. Conclusion

In conclusion, our study demonstrates that the health professionals surveyed perceive the relationship between climate crisis and health as an urgent issue and are aware of numerous impacts of the climate crisis on health. Respondents largely indicate that health professionals should advocate for climate action to ensure long-term health. Nevertheless, the majority has not yet mentioned the connection in their daily clinical work. The primary obstacles preventing interviewees from carrying out climate action during their work include a lack of time, materials and information. In private, however, the study participants are mainly already willing to engage in climate-protecting behaviour such as active mobility and a plant-based diet. There is a need for strengthening the climate-specific health literacy of health professionals and for the establishment of concepts that make climate-sensitive health advice possible despite the lack of time. In practice, a broad implementation of education on climate crisis and health and planetary health is needed, for example based on existing proposals. These actions underscore, the critical role of health professionals during the climate crisis, promoting both healthy and sustainable societies.

## Data availability statement

The datasets presented in this article are not readily available because of privacy restrictions. Requests to access the datasets should be directed to the corresponding author.

## Author contributions

LA, LR, ML, CB, JS, AW, and CJ contributed to the conception and design of the study and directly participated in interpretation of the results, provided critical comments to the manuscript and revised the text. LA and LR provided the questionnaire. LA performed the data collection and data preparation, performed the statistical analysis and data presentation, and wrote the first draft of the manuscript. CJ and AW supervised the project. All authors contributed to the article and approved the submitted version.
