# Supplementary material for: Climate-specific health literacy in health professionals: an exploratory study
Source: Front Med (Lausanne). 2023 Oct 20;10:1236319. doi: 10.3389/fmed.2023.1236319 (PMC10622978; doi:10.3389/fmed.2023.1236319)
Supplement: Supplementary file 1 [file Data_Sheet_1.PDF]

Supplementary Material

Climate-specific health literacy in health professionals: an exploratory study

Lorenz Albrecht[1]*, Lydia Reismann[1], Michael Leitzmann[1], Christine Bernardi[2], Julia von Sommoggy[2], Andrea Weber[1] † , Carmen Jochem[1] †

[1] Department of Epidemiology and Preventive Medicine, University of Regensburg, Regensburg, Germany

[2] Department of Epidemiology and Preventive Medicine/Medical Sociology, University of Regensburg, Regensburg, Germany

*** Correspondence:**

Corresponding Author: Lorenz Albrecht, Lorenz.albrecht@stud.uni-regensburg.de

Questionnaire

On climate-specific health literacy

Universitätsklinikum Regensburg

Dear participant,

by completing this questionnaire, you are supporting a research **project** on **climate-specific health literacy and thereby making an important contribution to sustainable medicine and long-term health!**
Participation is anonymous. For questions, please contact: planetare.gesundheit@ukr.de

Demographic characteristics

1	**Please select your gender and enter your age!**<br>☐ Male<br>☐ Female _______ Years<br>☐ Diverse
2	**What is your profession?**<br>☐ Physician<br>☐ Nurse
3	**What medical specialty would you most closely associate yourself with?**<br>☐ Internal Medicine ☐ Dermatology<br>☐ Surgery ☐ Radiation/ Nuclear Medicine<br>☐ Pediatrics ☐ Ear/ Nose/ Throat Medicine<br>☐ Anesthesia ☐ Ophthalmology<br>☐ Neurology

General information on climate awareness

The following questions each relate to the **general understanding of** climatic changes.

4	**What do you see as a particularly big challenge of climate change?** (Multiple selection possible)<br>☐ Temperature rise<br>☐ Melting of glaciers and sea level rise<br>☐ Extreme weather events<br>☐ Natural disasters<br>☐ Water scarcity or preservation of clean water<br>☐ Loss of biodiversity<br>☐ Economic crises and social inequality<br>☐ Problems in the global food supply<br>☐ Spread of infectious diseases<br>☐ None of the options listed here

Questionnaire
On climate-specific health literacy

Universitätsklinikum Regensburg

5	**How would you rate your level of knowledge regarding the general consequences of climate change?**	**1** very good	**2** good	**3** medium	**4** rather low	**5** very low
		☐	☐	☐	☐	☐

6	**How relevant does climate change seem to you at the moment?**	**1** very urgent	**2** import ant	**3** medium	**4** rather unim-portant	**5** absolutely collateral
		☐	☐	☐	☐	☐

7	**Do you think that your own (consumption) behaviour has an impact on climate change?**	**1** yes absolutely	**2** rather yes	**3** neither nor	**4** rather no	**5** not at all
		☐	☐	☐	☐	☐

8	**In your everyday life, do you make a conscious effort to keep your ecological footprint (i.e. your climate-damaging emissions) low?**	**1** yes absolutely	**2** rather yes	**3** neither nor	**4** rather no	**5** not at all
		☐	☐	☐	☐	☐

9	**How ready are you...?**	**1** very ready	**2** rather ready	**3** neither nor	**4** rather not	**5** No willingness
	... to use a bike/e-bike in everyday life instead of a fuel-consuming car?	☐	☐	☐	☐	☐
	... to use public transport (e.g. bus, train) for travel - instead of the car or plane?	☐	☐	☐	☐	☐
	... to get by in everyday life predominantly without meat and sausage products? (e.g. through a vegetarian diet)	☐	☐	☐	☐	☐
	... to get by predominantly without animal products in everyday life? (e.g. through a vegan diet)	☐	☐	☐	☐	☐
	... to get involved in sustainability and environmental protection in your environment or	☐	☐	☐	☐	☐
	... pay higher prices for products that are less harmful to the environment? (e.g. with organic certification)	☐	☐	☐	☐	☐

Questionnaire
On climate-specific health literacy

Universitätsklinikum Regensburg

10	**Does your willingness to implement the environmentally friendly measures described in question 6 increase if you ...**	**1** yes absolutely	**2** rather yes	**3** neither nor	**4** rather no	**5** not at all
	... know people in your environment who are active in a similar way?	☐	☐	☐	☐	☐
	...you implement these activities together with others?	☐	☐	☐	☐	☐
	... you know that you can strengthen your own health with it?	☐	☐	☐	☐	☐
	... you know that you can strengthen the health of the planet with it?	☐	☐	☐	☐	☐

Climate change and health

The following questions each refer to **health effects of** climatic changes.

11	**Do you consider climate change to be the cause of global health problems?** e.g. air pollution, food shortages, pandemics	**1** very likely	**2** likely	**3** neither nor	**4** rather no	**5** not at all
		☐	☐	☐	☐	☐
12	**Do you see climate change as a risk factor to your own (long-term) health?** e.g. infectious diseases, skin cancer, heat stress	**1** very likely	**2** likely	**3** neither nor	**4** rather no	**5** not at all
		☐	☐	☐	☐	☐
13	**Do you see climate change as a risk factor for your patients' health?** e.g. infectious diseases, skin cancer, heat stress	**1** very likely	**2** likely	**3** neither nor	**4** rather no	**5** not at all
		☐	☐	☐	☐	☐

Questionnaire
On climate-specific health literacy

Universitätsklinikum Regensburg

14	**What health consequences of climate change have you already heard about?** (multiple selection possible)<br><br>☐ Heat shock/heat stress<br>☐ Cardiovascular problems<br>☐ Increasing allergies<br>☐ Psychological complications (e.g. due to natural disasters)<br>☐ Respiratory complaints (e.g. due to air pollution).<br>☐ Increasing deficiency and undersupply worldwide<br>☐ Changing spread of infectious diseases<br><br>☐ I am well informed about these and other health consequences of climate change.<br><br>☐ I have not yet heard that climate change can have a negative impact on health.

Climate change mitigation in the healthcare system and climate-sensitive health advice

The following questions each refer to the **potential of the health system and medical staff for climate protection measures**.

15	**Which of the following measures do you think should be taken to reduce greenhouse gas emissions (e.g. CO_2) in the health system?**<br>(Multiple selection possible)<br><br>☐ Use of regenerative raw materials in medical devices<br>☐ Focus on sustainability in the education and training of medical staff<br>☐ Climate-friendly optimisation in the area of energy/electricity supply<br>☐ Climate-friendly measures in catering<br>(e.g. through more organic, regional, vegetarian or vegan meal options)<br>☐ Sustainable construction of health care facilities<br>☐ Sustainable mobility concepts for employees and patients to travel to and from healthcare facilities<br>☐ None of the measures, as the health system should focus on other concerns.<br>☐ Other measures:

Questionnaire

On climate-specific health literacy

Universitätsklinikum Regensburg

16	**Should medical staff be committed to climate protection to ensure long-term health?**	**1** yes definitely; *sustainability is important for health*	**2** rather yes	**3** neither nor	**4** rather no	**5** not at all; *physicians/ nurses have other tasks*
		☐	☐	☐	☐	☐

17 **In what way do you think medical staff should be committed to sustainability and the preventive safeguarding of health**?
(multiple selection possible)

- ☐ Education of patients by medical staff
- ☐ Conduct and disseminate targeted information campaigns (e.g. on concrete ways for employees and patients to protect the climate and thus their health in the long term).
- ☐ Make political efforts to reduce greenhouse gases
- ☐ Promote and conduct research on climate change and health
- ☐ Reducing plastic and disposable products in medical facilities
- ☐ Attending further training (e.g. for current knowledge/research on more sustainable products).
- ☐ None of these measures, as medical staff have other duties.
- ☐ Other:

18	**Do you see an opportunity for yourself to raise awareness and educate patients about climate change and health?**	**1** yes definitely	**2** rather yes	**3** neith er	**4** rather no	**5** not at all
		☐	☐	☐	☐	☐

19	**Have you ever mentioned the topic of climate change in connection with health?**	☐ yes	☐ no

20 **What would help you to educate about this topic even better**?

- ☐ Information about the scientific background of this topic
- ☐ Materials that I can use to explain the topic
- ☐ Time to be able to address the issue in my daily work
- ☐ Possibility of attending training courses on climate change and health
- ☐ Possibility of attending training courses on the topic of climate communication
- ☐ Other:

21	**Do you think you can promote a change towards a more sustainable climate-sensitive society through your work?**	**1** yes definitely	**2** rather yes	**3** neith er nor	**4** rather no	**5** not at all
		☐	☐	☐	☐	☐

Thank you very much for your participation!
